# Assessing Trait Covariation and Morphological Integration on Phylogenies Using Evolutionary Covariance Matrices

**DOI:** 10.1371/journal.pone.0094335

**Published:** 2014-04-11

**Authors:** Dean C. Adams, Ryan N. Felice

**Affiliations:** 1 Department of Ecology, Evolution, and Organismal Biology, Iowa State University, Ames, Iowa, United States of America; 2 Department of Biological Sciences, Ohio University, Athens, Ohio, United States of America; Midwestern University & Arizona State University, United States of America

## Abstract

Morphological integration describes the degree to which sets of organismal traits covary with one another. Morphological covariation may be evaluated at various levels of biological organization, but when characterizing such patterns across species at the macroevolutionary level, phylogeny must be taken into account. We outline an analytical procedure based on the evolutionary covariance matrix that allows species-level patterns of morphological integration among structures defined by sets of traits to be evaluated while accounting for the phylogenetic relationships among taxa, providing a flexible and robust complement to related phylogenetic independent contrasts based approaches. Using computer simulations under a Brownian motion model we show that statistical tests based on the approach display appropriate Type I error rates and high statistical power for detecting known levels of integration, and these trends remain consistent for simulations using different numbers of species, and for simulations that differ in the number of trait dimensions. Thus, our procedure provides a useful means of testing hypotheses of morphological integration in a phylogenetic context. We illustrate the utility of this approach by evaluating evolutionary patterns of morphological integration in head shape for a lineage of *Plethodon* salamanders, and find significant integration between cranial shape and mandible shape. Finally, computer code written in R for implementing the procedure is provided.

## Introduction

A major goal in evolutionary biology is to understand patterns of morphological covariation in biological forms. Advances in geometric morphometrics provide a detailed quantification of two- and three-dimensional shape diversity [1], [2], and investigations into the functional, genetic, and developmental causes of morphological variation result in hypotheses regarding how trait covariation evolves. Patterns and magnitudes of morphological trait covariation are referred to as *morphological integration* [3], [4], or the extent to which traits, or sets of traits, covary [5]–[8]. Because organisms must operate as an integrated whole, changes in some traits are accompanied by changes in other traits that are linked to them through common functional activities, developmental pathways, or genetic linkages and pleiotropy [9]. Over time this results in correlated evolution and thus covariation between traits. At the evolutionary level, deciphering how and why traits covary is critical for understanding the mechanisms that explain how morphological variation and covariation evolves [10], [11].

Over the past several years an increasing number of studies have investigated patterns of morphological integration and covariation in numerous traits and across a wide variety of taxa [5], [12]–[19]. Most have characterized morphological trait covariation within species, although some studies have also compared within-species patterns across multiple taxa [20]–[23] and others have examined interspecific patterns [24]–[28]. In such cases, understanding the evolution of morphological covariation requires an explicit macroevolutionary perspective, where patterns of covariation and integration are characterized across species. Here, the phylogenetic relationships among taxa must be considered, as species are not evolutionarily independent of one another [29]–[31]. To this end, the evolutionary associations between pairs of traits have been examined phylogenetically using elements in the evolutionary covariance matrix [32]. However, because hypotheses of morphological integration are typically interested in the degree of covariation between sets of traits, a pairwise trait approach is insufficient, as pairwise correlations do not capture the covariation between sets of traits treated as separate blocks of characters (much like Pearson’s correlation coefficient does not fully capture the information described by R^2^ in multiple regression). Thus, to evaluate patterns of morphological integration phylogenetically, alternative procedures must be used. Several studies have utilized phylogenetic regression to assess morphological covariation between two sets of traits of while accounting for phylogenetic non-independence (e.g., landmark configurations of face versus the braincase, or the ramus versus the alveolar region of the mandible) [23], [25], [26], [33]. Alternatively, analyses may be performed on the set of phylogenetic independent contrast scores obtained for the two sets of traits [24], [34], [35].

One approach commonly used to characterize ahistorical patterns of morphological integration between blocks of traits is partial least squares (PLS). Partial least squares is a statistical procedure that quantifies the degree of covariation between sets of variables, based on the overall trait covariance matrix [see 5,36,37]. PLS can be used to assess patterns of covariation between sets of morphological data (as in studies of morphological integration) or between a set of morphological data and another dataset (such as diet or environmental data). One advantage of this approach is that neither set of variables is assumed to be dependent on the other, as in regression analyses [5], [37]. This makes PLS a particularly useful tool for assessing the relationship between sets of traits that are hypothesized to covary but for which there is no *a priori* directional relationship posited between them.

Recently, patterns of morphological integration were evaluated in an explicit phylogenetic framework by evaluating covariation between sets of phylogenetic independent contrasts (PIC) for two sets of phenotypic traits [24], [34] using PLS and other approaches. However, because there is a direct statistical relationship between PIC and phylogenetic generalized least squares methods (PGLS) [30], [38], [39], one may also consider using the evolutionary covariance matrix from PGLS directly as the basis for evaluating phylogenetic morphological integration. Indeed, several authors have mentioned this as a possibility [24], [40], but to date none have demonstrated the equivalency (shown below). However, while PGLS- and PIC-based regression analyses provide identical statistical results under a Brownian motion evolutionary model [30], [38], [39], there are some advantages to implementing the PGLS-based approach for assessing morphological covariation in a phylogenetic context. First, PGLS approaches may be suitable for use under a variety of evolutionary models, including Brownian motion, Ornstein-Uhlenbeck, and models describing other evolutionary processes [41], [42]. Additionally, PIC assumes a completely bifurcating tree whereas PGLS may be used with trees containing polytomies [38].

For these reasons, examining phylogenetic morphological integration using this PGLS-based algorithm should be more broadly applicable than the previously published PIC-based methods [24], [34]. However, to date the full analytical procedure for implementing this approach has not been elucidated. Additionally, the statistical properties of methods that assess morphological integration in a phylogenetic context have not yet been explored. In this paper we outline an approach for estimating the strength of morphological integration in a phylogenetic context based on the evolutionary covariance matrix obtained from phylogenetic generalized least squares. Using computer simulations we show that statistical tests based on the approach display acceptable Type I error and appropriate statistical power for detecting phylogenetic morphological integration between blocks of traits. We then present a biological example assessing morphological integration in *Plethodon* salamanders demonstrating the utility of the method. Computer code written in R for implementing the procedure is also provided.

## Materials and Methods

### Estimating Phylogenetic Morphological Integration and Covariation Using Partial Least Squares

Partial least squares is used to assess covariation between subsets of data by utilizing a covariance matrix of the complete data. First, a *p*×*p* covariance matrix is constructed from an *N*×*p* data matrix (**Y**) containing the *p* phenotypic values for each of *N* specimens. In this case, **Y** represents two subsets (blocks) of variables, **Y_1_** and **Y_2_** (for generalizations to three sets of variables see: [5]). Thus, the phenotypic covariance matrix may be expressed as the partitioned matrix:
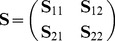
(1)where **S_11_** and **S_22_** describe the covariance within blocks **Y_1_** and **Y_2_** respectively, and 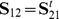 represents the covariation between blocks. Next, **S_12_** is decomposed using singular-value decomposition: 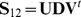 where the resulting **U** and **V** matrices contain linear combinations of variables for each block **Y_1_** and **Y_2_**. **D** is a diagonal matrix where each diagonal element contains the covariance between corresponding pairs of vectors in **U** and **V** [36], [37]. The largest value in **D** corresponds to the highest covariation between linear combinations of **Y_1_** and **Y_2_**. This may be found by multiplying (projecting) the original data (**Y_1_** and **Y_2_**) onto the vectors of linear combinations (**U** and **V** respectively) to obtain PLS scores, from which the maximal covariation between blocks may be calculated. One can also obtain the correlation between **Y_1_** and **Y_2_** using these PLS scores, which may then be used to statistically assess the association between **Y_1_** and **Y_2_** using permutation procedures [37], [43].

At a macroevolutionary scale, patterns of morphological integration are assessed from phenotypic data obtained from set of *N* species related by a phylogeny. To statistically evaluate patterns of evolutionary covariation in a phylogenetic context, one first requires a model that describes the evolutionary process that generated the data. Like many approaches, we utilize a Brownian motion model [29],[44]–[46]. Here, evolutionary changes in a single trait along the phylogeny are independent from time step to time step, with an expected displacement of zero and a variance among species (σ^2^) that accumulates proportional to time. For multivariate data, evolutionary change corresponds to a shift in the position of a species in a multivariate trait space whose axes correspond to trait dimensions. This process is described by an evolutionary covariance matrix (**R**) whose diagonal elements represent the expected variation for each trait dimension (σ^2^), and whose off-diagonal elements express the evolutionary covariation in changes between trait dimensions [47], [48]. Analytically this evolutionary covariance matrix may be estimated as:
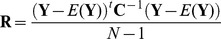
(2)where **Y** is a *N*×*p* matrix of phenotypic values for the *N* species, *E*(**Y**) is a *N*×*p* matrix where each row contains the multivariate phylogenetic mean found at the root of the phylogeny 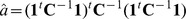, **1** is a *N*×1 column vector of ones, and **C** ^−1^ represents the inverse of the *N*×*N* phylogenetic covariance matrix describing the evolutionary relationships among species [30], [39], [49], [50]. Note that equation (2) describes the unbiased estimate of **R**, as the denominator is *N*-1 (for discussion see [30]).

In phylogenetic comparative biology, numerous procedures estimate the evolutionary covariance matrices as the starting point for evaluating hypotheses of changes in evolutionary rates among traits [48], [50]–[55] and shifts in trait covariances across the phylogeny [32], [48]. For assessing patterns of phylogenetic morphological integration, the evolutionary covariance matrix is first obtained. Next, **R** is represented as a partitioned matrix describing the evolutionary covariation within and between two sets of variables **Y_1_** and **Y_2_**:
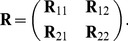
(3)A singular-value decomposition of **R_12_** is then performed: 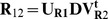 to obtain evolutionary linear combinations (**U_R1_** and **V_R2_**) for the two sets of variables.

To estimate the evolutionary correlation between the blocks of phenotypic data, **Y_1_** and **Y_2_**, projection scores on the first column of **U_R1_** and **V_R2_** are obtained. However, because **U_R1_** and **V_R2_** represent linear combinations from the evolutionary covariance matrix that takes phylogeny into account the original phenotypic data in **Y** must also be expressed phylogenetically prior to projection onto these axes. This is accomplished through phylogenetic transformation, as described in [30], [56]. First, the transformation matrix **D** is found from an eigen-decomposition of the phylogenetic covariance matrix: 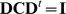. Next, the original phenotypic data, **Y**, are projected onto **D**, thereby transforming them by the phylogeny:
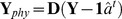
(4)To calculate evolutionary PLS scores, the two blocks of phylogenetically corrected data, **Y_1_**_***phy***_ and **Y_2_**_***phy***_, are multiplied by the first pair of vectors in **U_R1_** and **V_R2_**. From these the evolutionary correlation between the two blocks of data, **r** ***_PLS_***, is found by the correlation between the two vectors of PLS scores. Finally, the significance of this correlation, ***P_rand_***, may be assessed using phylogenetic permutation, where the phenotypic values for all species for one block (e.g., **Y_2_**) are permuted on the tips of the phylogeny, correlation scores are obtained from the permuted datasets, and these are compared to the observed value (for similar procedures see: [24], [57], [58]). Computer code written in R for implementing the approach is found in File S1.

### Statistical Performance of the Approach

The procedure specified above provides a logical means of characterizing evolutionary patterns of morphological integration in a phylogenetic context (for alternative formulations see [24], [26]). However, to date, none of the proposed tests of phylogenetic morphological integration have been evaluated in terms of their Type I error or statistical power (though the power of methods based on the RV coefficient are known to be sensitive to sample size: [59]). To alleviate this shortcoming, we examine the statistical performance of the hypothesis test proposed here, using a series of computer simulations. Simulations were conducted on a series of random phylogenies which differed in the number of taxa (*N* = 16, 32, 64, 128). For each simulation, the number of species was first selected and a random phylogeny was generated. Next, the total number of trait dimensions was selected (*p* = 6, 8, 10, 16, 20, 30), which were divided equally between two blocks: **Y_1_** and **Y_2_**. To simulate phenotypic data, an initial *p*×*p* covariance matrix was constructed. Simulations assumed isotropic error (σ^2^ = 1.0) for all trait dimensions, and these values were treated as the diagonal elements of the *p*×*p* covariance matrix. For the off-diagonal elements, covariation between trait dimensions was varied depending on simulation conditions. Simulations evaluating type I error rates used no initial association between trait dimensions (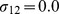), while simulations evaluating statistical power used increasing levels of covariation between trait dimensions (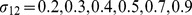). Phenotypic data were then obtained by simulating multi-dimensional traits following a Brownian motion model of evolution. Thus, the following procedure was used to generate 1000 data sets: 1) generate a random phylogeny, 2) generate an initial covariance matrix, 3) simulate data. For each dataset, the degree of morphological integration was then estimated using the procedure described above, and was evaluated statistically using phylogenetic permutation. For all simulations, when the initial covariation between sets of traits was greater than zero (i.e., when 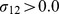), the proportion of significant results (out of 1000) provides an estimate of the statistical power of the test for that level of input covariation. Likewise, when the initial covariation between sets of traits was equal to zero (when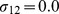), the proportion of significant results (out of 1000) provides an estimate of the Type I error rate of the test when no input covariation is provided.

#### Simulation results

**Figure 1:**
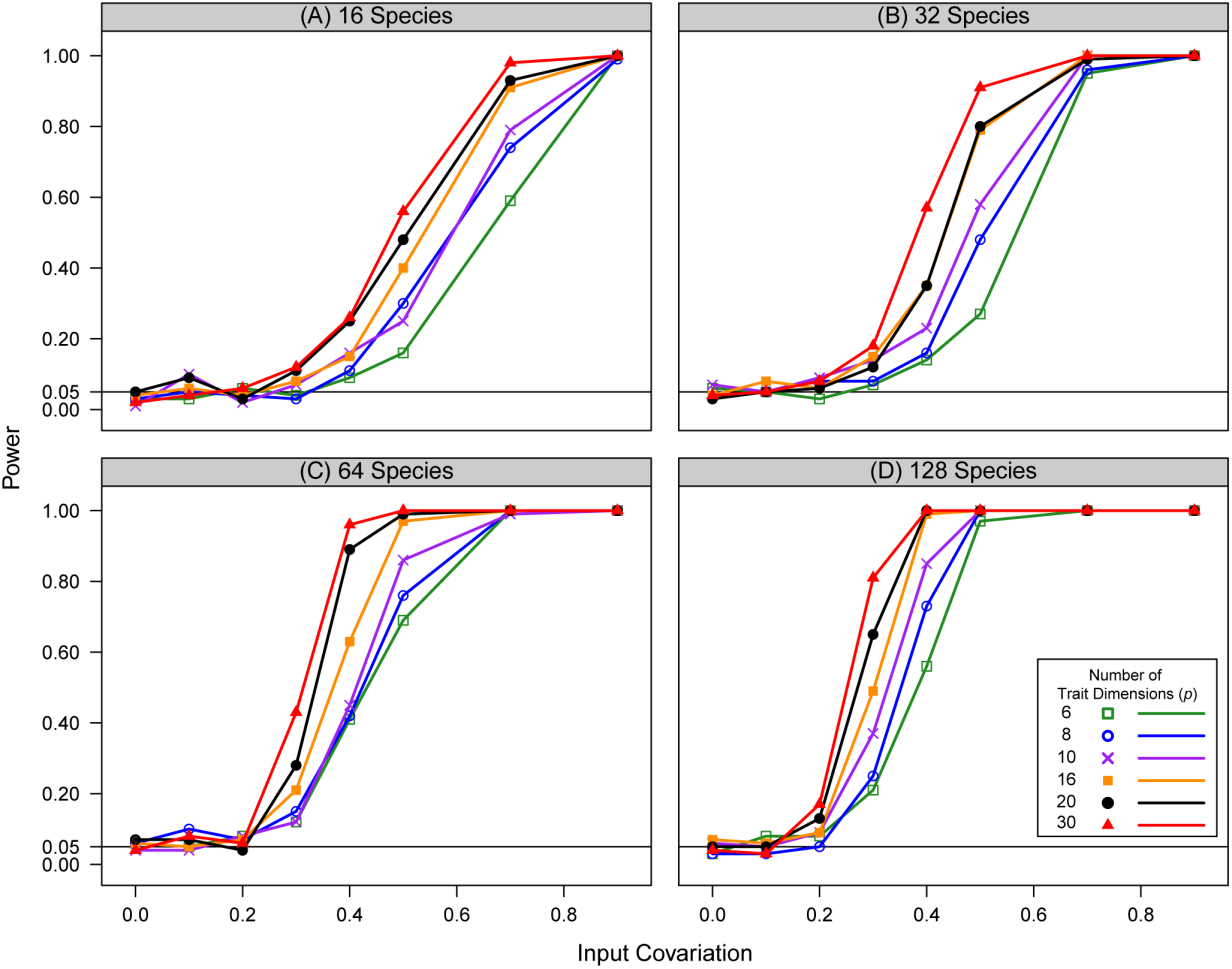
Type I error and statistical power of approach. Simulation results for type I error and statistical power of hypothesis testing procedures evaluating phylogenetic morphological integration. Type I error is found as the first point on each curve, where the input covariation between X and Y was 0.0 (see text). The horizontal line represents the expected type I error rate of 5%. Data were simulated under a Brownian motion model of evolution on randomly generated phylogenies containing: A) 16 species, B) 32 species, C) 64 species, and B) 128 species. Curves for increasing numbers of trait dimensions are shown.

For all simulations, hypothesis tests of phylogenetic morphological integration displayed appropriate Type I error rates near the nominal value of α = 0.05. This pattern remained consistent across the range of trait dimensionality examined in this study, and was consistent across the range of species richness evaluated here (Fig. 1). This finding is important, as it implies that the Type I approach developed here is relatively robust to sample size and trait dimensionality. This is in contrast to alternative methods of assessing morphological integration based on the RV coefficient, which lose power as sample size decreases [59].

In addition, the statistical power of tests evaluating phylogenetic morphological integration increased as the degree of covariation between **Y_1_** and **Y_2_** increased. This implied that the approach is better capable of detecting patterns of morphological integration as the strength of that signal is large. As expected, for the same number of trait dimensions, statistical power increased as the number of taxa examined increased (viewed across panels in Fig. 1). For example, when *p* = 10 there is considerably higher power to detect integration between blocks of traits when examining trends across 32 species as compared to just 16 species; and still more power to detect trends with a higher number of taxa. Thus, as with other phylogenetic comparative approaches utilizing a larger number of species is desirable.

Surprisingly, statistical power also increased with increasing numbers of trait dimensions. Thus, for a given number of taxa, the approach is better able to detect trends for traits of higher dimensionality than for traits represented by fewer dimensions. Similar findings were recently shown for methods evaluating multivariate rates of evolution on phylogenies [52], where power increased with increasing dimensionality. This result implies that measuring more complex traits does not impinge on one’s ability to evaluate morphological integration in high-dimensional multivariate data, as the procedure described here is capable of detecting these patterns. Similar results were obtained on phylogenies with different numbers of taxa (Fig. 1). Overall these simulations reveal that hypothesis tests of phylogenetic morphological integration and based on the evolutionary covariance matrix have appropriate Type I error and statistical power, and thus provide a useful means of detecting morphological integration and covariation in a phylogenetic context for high-dimensional phenotypic datasets.

### A Biological Example

As a biological example of the approach described here we evaluate the degree of phylogenetic morphological integration in the skulls of *Plethodon* salamanders. Vertebrate skulls are perhaps the most intensively studied anatomical structure in terms of morphological integration, and considerable evidence suggests that morphological integration in vertebrate skulls is displayed in a wide variety of taxa [5], [12], [15], [17], [24], [34], [60], [61]. The pervasive degree of skull integration is due to the fact that the component parts of the vertebrate skull share common developmental pathways, are under common functional demands, and thus evolve jointly with one another [62], [63]. In *Plethodon* salamanders, the head serves an important role during aggressive encounters, with the mandible and cranium operating together. Here, agonistic interactions begin with a series of visual displays between individuals [64], but these often escalate into direct attacks through biting [65]–[67]. Additionally, the head is also important for securing and immobilizing prey during foraging [43], [68]–[69]. Thus, it may be expected that cranial shape and mandible shape will be highly integrated in *Plethodon,* although this hypothesis has never been empirically examined. Further, because head shape exhibits a strong genetic component [70] it is thus reasonable to evaluate whether selection has driven the evolution of integration at the interspecific level.

**Figure 2:**
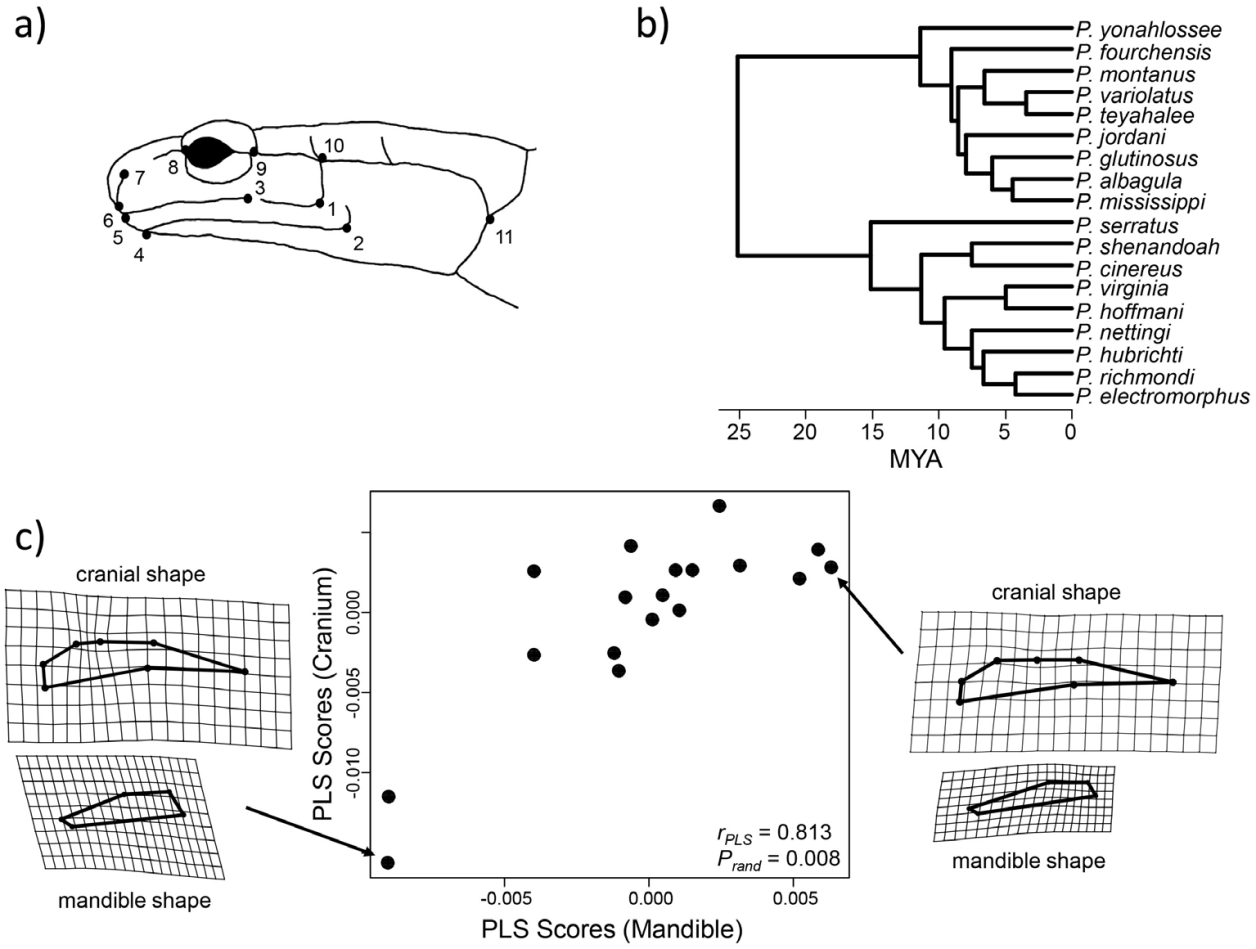
Data summary and analytical results evaluating morphological integration in *Plethodo* salamanders. a) Positions of 11 anatomical landmarks used to quantify head shape in *Plethodon* salamanders (image from [72]). b) Fossil-calibrated molecular phylogeny displaying the estimated phylogenetic relationships among the species of *Plethodon* examined here. c) Plot of scores along the first axis for mandible shape versus cranial shape found from a partial least squares analysis of the evolutionary covariance matrix (**R**). Thin-plate spline deformation grids for the representing the extreme forms along each axis are shown (magnified by 2X).

To test this hypothesis we quantified head shape from 691 adult salamanders from 18 species of *Plethodon* for which data were available (data from [71]–[76], see Table 1). Head shape was quantified using geometric morphometric methods [2], [77]. First, 11 landmarks were digitized from images of the left-lateral side of each head (Fig. 2a). Next, the position of the jaw was standardized relative to the skull by rotating the jaw to a common articulation angle among specimens [78]. Specimens were then aligned using a Generalized Procrustes analysis [79], and a set of shape variables were obtained for each specimen (Procrustes tangent coordinates). Both the cranium and mandible were superimposed simultaneously to take into consideration the relative size differences of the two structures (for discussion see [62]). The mean head shape was then calculated for each of the 18 species. We recognize that the use of species means does not allow within-species variation to be evaluated [24]. However, unlike likelihood-based PGLS approaches [56], methods for evaluating the effects of intraspecific variation in the context of phylogenetic partial least squares have not yet been developed, and are outside of the scope of the present paper.

**Table 1:** Number of adult specimens per species used in this study.

*Species*	*N*	*Species*	*N*
*Plethodon albagula*	23	*Plethodon montanus*	25
*Plethodon cinereus*	60	*Plethodon nettingi*	26
*Plethodon electromorphus*	73	*Plethodon richmondi*	106
*Plethodon fourchensis*	15	*Plethodon serratus*	11
*Plethodon glutinosus*	25	*Plethodon shenandoah*	30
*Plethodon hoffmani*	123	*Plethodon teyahalee*	25
*Plethodon hubrichti*	26	*Plethodon variolatus*	25
*Plethodon jordani*	25	*Plethodon Virginia*	23
*Plethodon mississippi*	25	*Plethodon yonahlossee*	25

Shape data were obtained using geometric morphometric methods, as described in the text.

With these data, the degree of morphological integration between the mandible (landmarks 1–5) and the cranium (landmarks 6–11) was evaluated in a phylogenetic context. For this, a multi-gene time-calibrated molecular phylogeny for *Plethodon* was used as an estimate of the species-level relationships ([80]: Fig. 2b). Phylogenetic morphological integration was then mathematically characterized using the method described above, and statistical significance was determined using phylogenetic permutation. In addition, phylogenetic morphological integration was also evaluated using a procedure based on phylogenetic independent contrasts [24], as implemented in MorphoJ [81]. Patterns of integration were then visualized using thin-plate spline deformation grids of both the cranium and mandible for exemplar specimens. All analyses were performed in R 3.0.1 (R Development Core Team [82]) using routines in the library geomorph [83], [84], and new routines written by the authors (File S1).

#### Biological example results

Analyses indicated that there was significant phylogenetic morphological integration between the cranium and mandible across species of *Plethodon* (r*_PLS_* = 0.813; *P_rand_* = 0.008). Among species, mandible shapes varied from relatively slender and elongated to more robust and laterally compressed (Fig. 2c). Additionally, salamanders displayed crania that were relatively more slender laterally to relatively laterally compressed (Fig. 2c). Taken together, the morphological integration between crania and mandibles was best described as a shift from individuals exhibiting relatively elongated mandibles and crania (on the positive side of the PLS axes) to individuals displaying relatively more robust mandibles with relatively more compressed crania (towards the negative side of the PLS axes). These analyses reveal that there is a significant degree of evolutionary morphological integration in the skulls of *Plethodon* found when the phylogenetic relationships among taxa are taken into consideration. Finally, a phylogenetic PLS analysis on the independent contrasts of shape confirmed that the approach developed here obtains identical results to those found using PIC-based methods under a Brownian motion model [24] for this biological example (r*_PLS_* = 0.813; *P_rand_* = 0.008).

## Discussion

A common feature of organisms is that some traits covary with one another, a pattern termed morphological integration. Patterns of morphological integration may be evaluated at differing levels of biological organization (developmental, ontogenetic, evolutionary), but when characterized across species, the phylogenetic relationships among taxa must be taken into account [24]. In this paper we outlined how the evolutionary covariance matrix obtained from phylogenetic generalized least squares may be utilized to estimate the degree of phylogenetic morphological integration between two sets of variables. Using computer simulations under Brownian motion we found that the approach has appropriate Type I error and statistical power. This represents a necessary in-depth assessment of the statistical performance of phylogenetically-informed PLS methods. This approach is shown to be capable of evaluating patterns of morphological integration across differing numbers of species and for traits of different dimensionality. We then examined morphological integration between the cranium and mandible in *Plethodon* salamanders, and found significant evolutionary integration between the two structures across taxa. We also provide an implementation of this approach in the R programming language. This application of phylogenetically-informed PLS may be used to evaluate not only morphological integration (correlation between two sets of landmark coordinates) but also correlation between morphology and other multivariate data, such as ecological or behavioral variables.

The method evaluated here provides a useful complement to other approaches that evaluate morphological integration using phylogenetic independent contrasts [24]. Indeed, we have demonstrated that when implemented properly, both methods will yield identical results for the same dataset (assuming a Brownian motion model of evolution), though complications from polytomies are avoided with the PGLS approaches. Further, at least in theory, the approach proposed here may be used to estimate the degree of phylogenetic morphological integration under a broader set of evolutionary models that characterize the tempo and mode of evolution under distinct evolutionary processes. However, to date, the statistical framework for evaluating alternative evolutionary models such as Ornstein-Uhlenbeck using highly multi-dimensional data has not yet been developed. Nonetheless, evaluating evolutionary morphological integration using the PGLS-derived evolutionary covariation matrix is a logical and flexible generalization of the PIC-based approach.

Finally, this procedure provides a tool for assessing macroevolutionary hypotheses regarding morphological integration. Phenotypic integration can influence patterns of variability and evolvability [10], [11], [23], [85], [86]. Accordingly, this concept has been hypothesized to be a factor in the evolution of morphological and lineage diversity, such as in adaptive radiations [23], [25], [26], [33]. To this end, the statistical framework herein represents a powerful method for estimating the degree of morphological integration and covariation in comparative datasets while taking in to account the non-independence of taxa.

## Supporting Information

File S1 **Computer Code for R.** The function estimates the degree of phylogenetic morphological covariation between two sets of variables using partial least squares. The observed value is statistically assessed using phylogenetic permutation, where data for one block are permuted across the tips of the phylogeny, an estimate of the covariation between sets of variables, and compared to the observed value.
(DOCX)
