# Supplementary material for: Assessing Trait Covariation and Morphological Integration on Phylogenies Using Evolutionary Covariance Matrices
Source: PLoS One. 2014 Apr 11;9(4):e94335. doi: 10.1371/journal.pone.0094335 (PMC3984176; doi:10.1371/journal.pone.0094335)
Supplement: File S1 — Computer Code for R. The function estimates the degree of phylogenetic morphological covariation between two sets of variables using partial least squares. The observed value is statistically assessed using phylogenetic permutation, where data for one block are permuted across the tips of the phylogeny, an estimate of the covariation between sets of variables, and compared to the observed value. (DOCX) [file pone.0094335.s001.docx]

**Supplemental File S1: Computer Code for R.** The function estimates the degree of phylogenetic morphological covariation between two sets of variables using partial least squares. The observed value is statistically assessed using phylogenetic permutation, where data for one block are permuted across the tips of the phylogeny, an estimate of the covariation between sets of variables, and compared to the observed value.

#The function below estimates the degree of phylogenetic morphological covariation between two

#sets of variables using partial least squares. The observed value is statistically assessed

#using phylogenetic permutation, where data for one block are permuted across the tips of the phylogeny,

#an estimate of the covariation between sets of variables, and compared to the observed value.

#To use this function, "block1" and "block2" should be 2- or 3-dimensional arrays of phenotypic data

#between which morphological integration will be assessed. "phy" should be an object of class phylo

#with tip labels corresponding to the rownames of the blocks of data.

Phylo.Morphol.PLS <-function(block1, block2, phy, iter=999){

library(ape)

library(geomorph)

if (class(phy) != "phylo")

stop("phy must be of class 'phylo.'") #confirm that the input phylogenetic tree is of class 'phylo'

# count the number of taxa and create a vector of the species names:

if(length(dim(block1)) == 3){

num.taxa.X<-dim(block1)[3]

namesX<-dimnames(block1)[[3]]}

else{ num.taxa.X<-nrow(block1)

namesX<-rownames(block1)}

if (is.null(namesX)){

stop("No specimen names in data matrix 1. please assign specimen names.") } #confirms that species names are present

if (length(match(phy$tip.label, namesX)) != num.taxa.X && length(phy$tip.label) < num.taxa.X)

stop("Tree is missing some taxa present in the data matrix") #confirm that there are equal numbers of taxa in the tree and the dataset

if (length(match(phy$tip.label, namesX)) != num.taxa.X && num.taxa.X < length(phy$tip.label))

stop("Tree contains some taxa not present in present in the data matrix") #confirm that there are equal numbers of taxa in the tree and the dataset

if (length(grep("-999", block1)) != 0) {

stop("Data matrix 1 contains missing values. Estimate these first(see 'estimate.missing').") } #no missing values are allowed

if (length(grep("-999", block2)) != 0) {

stop("Data matrix 2 contains missing values. Estimate these first(see 'estimate.missing').") }

if(length(dim(block2)) == 3){

num.taxa.Y<-dim(block2)[3]

namesY<-dimnames(block2)[[3]]}

else{ num.taxa.Y<-nrow(block2)

namesY<-rownames(block2) }

if (is.null(namesY)){

stop("No specimen names in data matrix 2. please assign specimen names") } #confirms that species names are present

if (is.null(namesX) == FALSE && is.null(namesY) == FALSE) {

mtch.A <- namesX[is.na(match(namesX, namesY))]

if (length(mtch.A) > 0) {

stop("Specimen names in data sets are not the same.") } #confirms that the species names match in both blocks of data

}

mtch.B <- namesX[is.na(match(namesX, phy$tip.label))]

if (length(mtch.B) > 0) {

stop("Taxa labels on tree and taxa matrix are not the same.")} #confirms that the species names match in the data and the phylogeny

if (length(dim(block1)) == 3){

x<-two.d.array(block1)}

else {x<-block1 }

if (length(dim(block2)) == 3){ y<-two.d.array(block2) }

else { y<-block2 }

data.all<-cbind(x,y) #combines the two blocks into a single data array

Nspec<-nrow(x) #counts the number of species

C<-vcv.phylo(phy,anc.nodes=FALSE) #finds the phylogenetic variance-covariance matrix for the input phylogeny

C<-C[rownames(y),rownames(y)] #sorts this VCV matrix to be in the same order as the data matrix

x<-x[rownames(y),] #makes sure that the taxa are in the same order in both blocks of data

invC<-solve(C) #compute inverse of C

one<-matrix(1,Nspec,1) #generates a vector with length=number of taxa of 1's

a<-t(t(one)%*%invC%*% data.all)*sum(sum(invC))^-1 #estimation of phylogenetic mean- the character values at the root of the phylogeny

R<- t(data.all-one%*%t(a))%*%invC%*%(data.all-one%*%t(a))*(Nspec-1)^-1 #generate evolutionary covariance matrix, R

R12<- R[1:dim(x)[2], (dim(x)[2] + 1):(dim(x)[2] + dim(y)[2])] #isolates that part of R which describes covariantion between blocks 1 and 2 only

pls <- svd(R12) #compute partial least squares of R12

U <- pls$u #weights for variables in block one

V <- pls$v #weights for variables in block two

eigC<-eigen(C)

D.mat<-solve(eigC$vectors %*% diag(sqrt(eigC$values)) %*% t(eigC$vectors)) #transformation matrix D

Phy.X<-D.mat%*%( data.all-one%*%t(a)) #Phy.X is the phylogenetically transformed data (equation 4 in the manuscript)

x.phy <- Phy.X[, c(1:dim(x)[2])] #the part of the transformed data corresponding to block 1

y.phy <- Phy.X[, c((dim(x)[2] + 1):(dim(x)[2] + dim(y)[2]))] #the part of the transformed data corresponding to block 2

XScores <- x.phy %*% U[, 1] #Compute Block 1 PLS scores

YScores <- y.phy %*% V[, 1] #Compute Block 2 PLS scores

pls.obs <- cor(XScores, YScores) #compute correlation between the two blocks

P.val <- 1

pls.val <- rep(0, iter)

#finally, resample the data and iterate the steps above.

for (ii in 1:iter) {

y.r <- y[sample(nrow(y)), ]

data.all.r<-cbind(x,y.r)

a.r<-t(t(one)%*%invC%*%data.all.r)*sum(sum(invC))^-1

R.r<- t(data.all.r-one%*%t(a.r))%*%invC%*%(data.all.r-one%*%t(a.r))*(Nspec-1)^-1

R12.r <- R.r[1:dim(x)[2], (dim(x)[2] + 1):(dim(x)[2] + dim(y.r)[2])]

pls.r <- svd(R12.r)

U.r <- pls.r$u

V.r <- pls.r$v

Phy.X.r<-D.mat%*%( data.all.r-one%*%t(a.r))

x.phy.r <- Phy.X.r[, c(1:dim(x)[2])]

y.phy.r <- Phy.X.r[, c((dim(x)[2] + 1):(dim(x)[2] + dim(y.r)[2]))]

XScores.r <- x.phy.r %*% U.r[, 1]

YScores.r <- y.phy.r %*% V.r[, 1]

pls.r <- cor(XScores.r, YScores.r)

pls.val[ii] <- pls.r

P.val <- ifelse(pls.r >= pls.obs, P.val + 1, P.val)

}

pls.val[iter + 1] = pls.obs

P.val <- P.val/(iter + 1)

lim.x <- c(min(XScores),max(XScores))

lim.y <- c(min(YScores),max(YScores))

X11()

hist(pls.val, 30, freq = TRUE, col = "gray", xlab = "PLS Correlation")

arrows(pls.obs, 50, pls.obs, 5, length = 0.1, lwd = 2)

X11()

plot(XScores, YScores, pch = 21, bg = "black", xlim=lim.x, ylim=lim.y, main = "PLS Plot", xlab= "Block 1 PLS Scores", ylab="Block 2 PLS Scores")

return(list("PLS Correlation" = pls.obs, pvalue = P.val, "Block 1 PLS Scores" = XScores, "Block 2 PLS Scores" = YScores))

}
